# Mapping Anxiety, Stress, Depression, Resilience and Happiness in the Adolescent Population: A Network Analysis and Comparison by Sex

**DOI:** 10.3390/ejihpe16020031

**Published:** 2026-02-19

**Authors:** Roger Angulo-Salas, Jonatan Baños-Chaparro, Geraldinne Ayala Garcilazo, Jeremy Yovani Juarez Medina, Delly Santos-Chuquispuma

**Affiliations:** 1 Faculty of Health Sciences, Universidad Católica Sedes Sapientiae, Lima 15046, Peru; rjangulo@ucss.edu.pe (R.A.-S.); geraldinnegarcilazo@gmail.com (G.A.G.); jeremiasjm73@gmail.com (J.Y.J.M.); 2 Academic Program of Psychology, Faculty of Health Sciences, Universidad Privada Norbert Wiener, Lima 15046, Peru; a2023100050@uwiener.edu.pe

**Keywords:** mental health, adolescent, psychological well-being, risk factors, protective factors

## Abstract

Background: Adolescence is a developmental window of heightened vulnerability to psychological distress, yet the interplay between pathology and protective factors remains understudied in a low-to-middle-income urban district in North Lima, Peru. This study examined the network structure of resilience, happiness, and mental health indicators in Peruvian adolescents to identify precise intervention targets. Methods: A sample of 559 adolescents (49.9% boys; *M*_age_ = 14.72, SD = 1.43) recruited from public secondary schools in Carabayllo, a low-to-middle-income urban district in North Lima, Peru, completed validated measures of resilience (CD-RISC-25), subjective happiness, and mental health (anxiety, depression, and stress). A Gaussian Graphical Model was estimated using non-regularized partial correlations. Node centrality, predictability, and network stability were assessed, and a Network Comparison Test evaluated structural differences by sex. Results: Anxiety, depression, and stress formed a tightly interconnected core, with the strongest edge between stress and anxiety. Among the psychological resources, self-regulation and external resources showed the highest centrality and predictability, followed by personal competence and tenacity. Happiness occupied a peripheral position but maintained a negative association with depression. The network demonstrated strong stability (CS = 0.75). No significant structural or global strength differences emerged between boys and girls. Conclusions: Findings challenge generic well-being approaches, revealing that happiness is a distal factor rather than a central buffer in this population. Instead, the network architecture suggests that interrupting the stress–anxiety loop and fostering self-regulation skills constitute the most effective pathways for school-based mental health protection, regardless of student gender.

## 1. Introduction

Adolescence is a critical developmental period characterized by profound biological, cognitive, and psychosocial changes that heighten both vulnerability to mental health problems and capacity for adaptation. This stage constitutes a sensitive developmental window in which increased neuroplasticity amplifies susceptibility to stress while also enabling adaptive responses to adverse and positive experiences (Sisk & Gee, 2022). As a result, adolescents are especially susceptible to engaging in risk behaviors and developing mood-related and psychological distress symptoms (Uccella et al., 2023).

Globally, adolescent mental health problems represent a substantial public health burden with significant consequences for academic, social, and personal development. It is estimated that one in seven adolescents aged 10–19 experiences a mental health problem, many of which remain unrecognized and untreated (Institute for Health Metrics and Evaluation [IHME], 2024). Evidence from the Global Burden of Disease study indicates sustained increases in anxiety and depressive disorders from 1990 to 2019, accounting for millions of disability-adjusted life years among adolescents (Piao et al., 2022). Consistent with this, poorer mental health has been associated with lower academic performance, whereas better psychological functioning is linked to more favorable educational outcomes (Chidoluo Vitus, 2024; Suvitha et al., 2023).

Within the spectrum of adolescent mental health, anxiety, depression, and stress are among the most prevalent and persistent manifestations of psychological distress. Recent estimates indicate that anxiety disorders affect approximately 4.1% of adolescents aged 10–14 and 5.3% of those aged 15–19, whereas depression affects 1.3% and 3.4% of these groups, respectively (Institute for Health Metrics and Evaluation [IHME], 2024). A growing body of research demonstrates that anxiety, depression, and stress frequently co-occur and share common patterns of psychological distress that contribute to their persistence over time (Higueras et al., 2022; Iannattone et al., 2024). Studies in Latin American educational contexts further reveal particularly high prevalence rates of these conditions, reinforcing the need for integrated analytical approaches (Martínez-Líbano & Yeomans-Cabrera, 2024).

Contemporary conceptualizations of mental health increasingly adopt a multidimensional perspective that integrates both psychological distress and positive psychological resources. From this standpoint, mental health is not merely the absence of psychopathology but encompasses varying levels of functioning and well-being (Kanougiya et al., 2024). Dual-continua models propose the coexistence of two related but distinct dimensions—psychological distress and mental wellbeing—suggesting that psychological resources may mitigate future mental health risks and promote more adaptive developmental trajectories (Bohlmeijer & Westerhof, 2021; Burns et al., 2022).

Within this framework, resilience has been consistently identified as a central protective factor during adolescence. Defined as the capacity to adapt effectively to adversity, resilience is associated with lower levels of anxiety, depression, and stress, as well as better adjustment to stressful events (Masten et al., 2021; Mesman et al., 2021; Wen et al., 2023). Longitudinal evidence further shows that higher baseline resilience predicts subsequent reductions in psychological distress, even in highly adverse contexts such as the COVID-19 pandemic (Shi et al., 2022). Meta-analytic results also show that interventions that focus on resilience have long-lasting positive effects on the mental health of teenagers (Pinto et al., 2021). From a network perspective, resilience should not be conceptualized as a unitary construct but rather as a constellation of interrelated capacities: while self-confidence and self-trust and personal competence and tenacity reflect relatively stable personal resources, internal resources and—particularly—self-regulation and external resources capture dynamic regulatory and contextual processes that are more likely to occupy central positions within psychological networks.

Similarly, happiness—conceptualized as subjective well-being encompassing affective experiences and life satisfaction—has been linked to lower psychological distress and greater psychological resources (Nima et al., 2024). Empirical studies demonstrate that higher happiness is associated with reduced anxiety, depression, and stress, as well as improved well-being in adolescents and young adults (Carreno et al., 2021; Milovanska-Farrington & Farrington, 2022). Recent research suggests that happiness may contribute to mental health by fostering more positive appraisals and enhancing coping capacities in response to environmental demands (Sotiropoulou et al., 2023).

Despite these advances, much of the existing literature relies on traditional latent variable models based on global scores, which assume that symptoms are manifestations of underlying constructs. Although informative, this approach obscures direct interactions among specific indicators (e.g., how stress directly activates anxiety), limiting the identification of precise intervention targets (Borsboom & Cramer, 2013). Moreover, most evidence derives from WEIRD populations (Western, Educated, Industrialized, Rich, and Democratic), leaving a significant gap regarding how the architecture of distress and resilience is organized among adolescents in low- and middle-income contexts, where structural stressors may shape psychological processes differently.

Although network models can, in principle, be estimated at the level of individual items or symptoms, the present study operates at the level of theoretically and psychometrically validated subscales. This decision reflects a trade-off between conceptual granularity and statistical stability. Item-level networks require enormous samples to ensure reliable edge estimation and often produce highly complex structures that are difficult to interpret and translate into practical recommendations. By contrast, modelling well-established dimensions of resilience, happiness, and psychological distress allows for a more stable and interpretable representation of the mental health system while still capturing direct associations among key psychological domains.

In response to these limitations, psychometric network analysis has emerged as a complementary framework that conceptualizes mental health as a system of directly interconnected variables rather than manifestations of latent entities (Epskamp & Isvoranu, 2022; Briganti et al., 2024). Network studies in adolescents consistently show that anxiety, depression, and stress occupy central, mutually reinforcing positions, whereas resilience may function as a structural buffer against symptomatology (Hu et al., 2025; Zhang et al., 2024; Li & Kwok, 2023; Wen et al., 2023). Nevertheless, studies integrating resilience, happiness, and mental health within a single network remain scarce, particularly in Latin American contexts and with explicit comparisons by sex.

In the Peruvian context, these dynamics may be further shaped by structural stressors typical of lower–middle-income urban settings, including socioeconomic inequality, academic pressure, family economic insecurity, and heterogeneous access to educational and mental health resources. Such conditions are likely to intensify the centrality of stress-related nodes (e.g., stress and anxiety) while increasing the functional relevance of regulatory capacities and external supports within the resilience system. Consequently, examining the network structure of mental health in Peruvian adolescents is not only a matter of representativeness but also of understanding how contextual constraints may reconfigure the relational architecture linking distress and protective resources.

Accordingly, the present study seeks to characterize the relational architecture linking resilience, happiness, and mental health (anxiety, depression, and stress) in Peruvian secondary school adolescents—an underrepresented population in the global literature. Rather than treating these constructs as independent or solely latent phenomena, we adopt a network perspective that conceptualizes mental health as a system of directly interacting variables. Grounded in dual-continua models of mental health and recent advances in network psychopathology, the following hypotheses were formulated to test theoretically driven predictions regarding (a) the centrality of distress symptoms, (b) the structural role of resilience dimensions, (c) the position of happiness, and (d) potential sex-related differences in network organization.

### Hypotheses

The present study tests the following hypotheses, drawing on dual-continua models of mental health and recent evidence from network psychopathology:

**H1.**  *Stress and anxiety will exhibit the strongest direct connection in the network.*

Rationale. Prior network studies consistently identify stress and anxiety as central, mutually reinforcing nodes within adolescent psychopathology, suggesting a core feedback loop that sustains distress and facilitates the spread of activation to other symptoms (Hu et al., 2025; Zhang et al., 2024).

**H2.**  *The self-regulation and external resources dimension of resilience will show the highest expected influence and predictability in the network.*

Rationale. Contemporary evidence indicates that regulatory capacities and access to external resources play a more critical role in adaptive functioning than static personal traits, positioning this dimension as a key structural buffer against psychological distress (Masten et al., 2021; Wen et al., 2023).

**H3.**  *Happiness will occupy a more peripheral position in the network relative to distress and resilience nodes.*

Rationale. Although happiness is negatively associated with psychopathology, theoretical and empirical work suggests that hedonic well-being may function more as an outcome of mental health rather than a proximal driver of symptom dynamics within psychopathological networks (Burns et al., 2022; Galderisi, 2024).

**H4.**  *The overall network structure will be invariant across sex.*

Rationale. Although prevalence of internalizing symptoms tends to differ by sex, emerging network evidence suggests that the underlying structural organization of distress is largely comparable between males and females, implying that differences are more likely due to symptom severity or activation thresholds rather than distinct network architectures (Sacco et al., 2024).

## 2. Materials and Methods

### 2.1. Participants

The study employed a cross-sectional, quantitative, and associative design. Participants were eligible for inclusion if they were adolescents regularly enrolled in the educational institution and present in the classroom at the time of data collection. Participants were recruited from public secondary schools located in North Lima, Peru, following a convenience sampling strategy.

The recruitment process followed a two-stage procedure. First, formal authorization was obtained from school principals and administrative authorities. Second, classrooms were selected within each grade level, and all students present in the selected classrooms were invited to participate.

Inclusion further required voluntary informed assent from the students, as well as written informed consent from their parents or legal guardians, in accordance with ethical guidelines for research involving minors. Students who declined to participate or withdrew from the study at any point were excluded. Additionally, exclusion criteria comprised the presence of physical, sensory, or cognitive difficulties that impeded adequate comprehension or completion of the assessment instruments, as well as the absence of signed informed consent from a legal guardian.

Data were collected using a paper-and-pencil questionnaire administered collectively in the classroom by trained researchers during regular school hours. The administration lasted approximately 30–40 min. Participants were informed about the voluntary nature of their participation, the anonymity of their responses, and the confidentiality of the data. No incentives were provided for participation.

Before data entry, all questionnaires were checked in situ by trained researchers to ensure that they were fully completed. Consequently, the final dataset did not contain missing values, and no imputation procedures were required.

The final sample consisted of 559 secondary school adolescents (49.9% male, *n* = 279; 50.1% female, *n* = 280), aged between 12 and 17 years (*M* = 14.72, *SD* = 1.43), who were enrolled from the first to the fifth year of secondary education (Table 1). Because a convenience sampling strategy was employed, the findings should be interpreted with caution when generalizing to adolescents from other regions (e.g., rural areas) or to clinical populations; nevertheless, the sample is suitable for the exploratory and structural aims of the present network analysis within comparable urban school contexts.

### 2.2. Sample Size and Power Analysis

To determine the sample size required for the planned Gaussian graphical model (GGM), a power analysis was conducted using Monte Carlo simulations implemented in the *powerly* package (Constantin et al., 2023), following methodological recommendations by Epskamp and Fried (2018). A true network consisting of eight nodes—anxiety, depression, stress, four dimensions of resilience, and happiness—was specified, assuming a global network density of 0.40, which was held constant across all simulations. Network estimation was performed using EBICglasso, and model performance was evaluated based on edge sensitivity.

The initial optimization phase indicated that approximately 432 participants were required to achieve a minimum sensitivity of 0.80 with a statistical power of 0.80. This estimate was subsequently validated through 1000 Monte Carlo replications, which confirmed that the sensitivity criterion was consistently met from a sample size of N = 443 onwards (power = 0.822; sensitivity at the 20th percentile = 0.846).

The final sample of the present study comprised 559 adolescents, substantially exceeding this validated threshold, thereby ensuring adequate statistical power and stable recovery of the underlying network structure.

### 2.3. Measures

#### 2.3.1. Demographic Information

The sociodemographic variables considered included sex (male/female), age, peer relationship, family relationship, study resources (yes/no), recreational activities (yes/no), and psychological treatment or counselling (yes/no).

#### 2.3.2. Connor–Davidson Resilience Scale (CDRS)

Resilience was assessed using the CDRS in its original 25-item version (Connor & Davidson, 2003), employing the South American adaptation validated in the Peruvian population, which demonstrates adequate psychometric properties and measurement invariance across sex (Dominguez-Cancino et al., 2022). The scale comprises four dimensions: Self-confidence and self-trust, internal resources, personal competence and tenacity, and self-regulation and external resources. Items are rated on a five-point Likert-type scale ranging from 0 (never) to 4 (almost always), with higher scores indicating greater levels of resilience. Because the CDRS is a copyrighted instrument, formal authorization for its use was obtained directly from the scale’s author. A licensing fee was paid for the right to use the instrument in this study, and the corresponding materials were provided by the author prior to data collection. In the present study, the scale exhibited an adequate four-factor factorial structure, with acceptable fit indices (CFI = 0.934; SRMR = 0.050; RMSEA = 0.061, 95% CI [0.056, 0.065]), as well as satisfactory reliability estimated using omega: self-confidence and self-trust (ω = 0.70), internal resources (ω = 0.69), personal competence and tenacity (ω = 0.70), and self-regulation and external resources (ω = 0.82).

#### 2.3.3. Subjective Happiness Scale (SHS)

Happiness was assessed using the SHS (Lyubomirsky & Lepper, 1999), in its four-item Spanish-adapted version, which demonstrates adequate psychometric properties and measurement invariance across sex (Extremera & Fernández-Berrocal, 2014). This instrument evaluates global perceptions of subjective happiness through statements involving self-evaluation and comparison with others. Items are rated on seven-point Likert-type scales, with response formats varying according to item content. The total score is obtained by calculating the mean of the items, with higher values reflecting greater subjective happiness. In the present study, the scale exhibited an adequate unidimensional factorial structure, with satisfactory fit indices (CFI = 0.99; RMSEA = 0.02, 95% CI [0.001, 0.092]; SRMR = 0.01), as well as acceptable reliability estimated using omega (ω = 0.72).

#### 2.3.4. Depression, Anxiety and Stress Scale–21 (DASS-21)

Mental health was assessed using the DASS–21 (Lovibond & Lovibond, 1995), in its version adapted and validated for the Peruvian population, which demonstrates adequate psychometric properties and measurement invariance across sex (Contreras-Mendoza et al., 2021). The instrument comprises 21 items distributed across three dimensions: depression, anxiety, and stress, each consisting of seven items. Responses are recorded on a four-point Likert-type scale ranging from 0 (did not apply to me) to 3 (applied to me very much). Scores for each dimension are obtained by summing the corresponding items, with higher scores indicating greater symptom severity. In the present study, the Peruvian version of the DASS-21 demonstrated an adequate three-factor factorial structure, with excellent fit indices (CFI = 0.954; SRMR = 0.054; RMSEA = 0.068, 95% CI [0.062, 0.074]), as well as satisfactory reliability estimated using omega: stress (ω = 0.80), anxiety (ω = 0.81), and depression (ω = 0.85).

All selected instruments have demonstrated validity in adolescent populations. Specifically, the CD-RISC-25 (Dominguez-Cancino et al., 2022) and DASS-21 (Contreras-Mendoza et al., 2021) have shown adequate psychometric properties in Peruvian adolescents. Furthermore, the Spanish Subjective Happiness Scale (Extremera & Fernández-Berrocal, 2014) was validated in samples explicitly including high school students (*M*_age_ = 16.69). Thus, applying these measures to 12–17-year-olds is grounded in prior developmental and cross-cultural evidence, independent of the statistical fit observed herein.

### 2.4. Statistical Analysis

Statistical analyses were conducted using RStudio software (version 4.3.2). In an initial phase, descriptive statistics such as means and standard deviations were calculated to summarize average scores. Prior to network construction, node redundancy was examined using the goldbricker function from the *networktools* package (Jones, 2021), identifying pairs of nodes with more than 25% topological overlap and applying a significance threshold of *p* = 0.05 (Hittner et al., 2003).

The estimation of the undirected network was conducted in three phases. In the first phase, the estimateNetwork function from the *bootnet* package was used to generate a non-regularized network model, employing the ggmModSelect algorithm with Spearman correlations, given their suitability for non-normally distributed data (Isvoranu & Epskamp, 2023). This algorithm selects the most appropriate Gaussian graphical model from 100 random models, guided by the extended Bayesian information criterion (EBIC).

The choice of a non-regularized GGM (ggmModSelect) was guided by both theoretical and statistical considerations. Regularized approaches such as EBICglasso are advantageous for high-dimensional settings and for controlling false-positive edges through shrinkage, but they may also introduce false negatives by attenuating small yet meaningful associations. In contrast, non-regularized model selection prioritizes edge recovery when the sample size substantially exceeds the number of nodes, as in the present study (N = 559; 8 nodes), allowing more accurate estimation of partial correlations and centrality metrics. This approach aligns with recent methodological recommendations for network psychometrics, which emphasize a trade-off between discovery (edge sensitivity) and caution (edge sparsity).

Non-regularized approaches are appropriate when the number of participants exceeds the number of nodes and when the aim is to explore edges and centrality within the network (Burger et al., 2023; Isvoranu & Epskamp, 2023; Williams et al., 2019). Network visualization was performed using the *qgraph* package, applying the Fruchterman–Reingold algorithm: nodes are represented as circles and conditional associations as lines, with positive connections shown in blue and negative connections in red. Line thickness and color intensity indicate the magnitude of the association (Epskamp et al., 2012; Fruchterman & Reingold, 1991).

In the second phase, both local and global network properties were examined. At the local level, expected influence (EI) was estimated using the centrality function from the *qgraph* package, which considers the direction of connections to determine each node’s overall relevance (Epskamp et al., 2012). Predictability was also assessed using the coefficient of determination (R^2^), calculated with the predict function from the *mgm* package, reflecting the extent to which a node can be predicted by its direct neighbors (Haslbeck & Waldorp, 2018, 2020). At the global level, three metrics were calculated: density (D), representing the average strength of connections between nodes; global transitivity (Cc; also referred to as the clustering coefficient), assessing the degree of clustering; and average shortest path length (APL), indicating efficiency in information propagation. The small-world index (S) was also computed, with values greater than 1 indicating a well-connected network with meaningful clustering structures, using the smallworldIndex function from the *qgraph* package (Epskamp et al., 2012; Isvoranu et al., 2022).

In the third phase, network accuracy and stability were evaluated using non-parametric bootstrap methods. A total of 1000 bootstrap samples were generated using the *bootnet* package, and 95% confidence intervals were calculated for each edge (Burger et al., 2023). To assess stability, a case-dropping subset bootstrap procedure was applied, re-estimating the network after each random removal. The correlation stability coefficient (CS) was calculated, indicating the maximum proportion of data that can be excluded without substantially affecting the stability of expected influence. A CS value greater than 0.25 was considered adequate (Burger et al., 2023; Isvoranu et al., 2022).

Finally, in the fourth phase, a comparative analysis was conducted between the network structures of females and males. Similarity between the two networks was assessed using Pearson correlation via the cor function. Subsequently, a permutation test was performed using the NCT function from the *NetworkComparisonTest* package, comparing two independent groups through 1000 random permutations to test the null hypothesis (van Borkulo et al., 2023). This evaluation was based on two indicators: the maximum statistic (M), which estimates the degree of global network structural similarity between groups, and the global strength difference (S_i_), which quantifies the weighted sum of absolute differences between connections across both networks (Isvoranu et al., 2022). Holm–Bonferroni correction was applied in both analyses, with differences considered significant at *p* < 0.05 (van Borkulo et al., 2023).

### 2.5. Ethical Considerations

The study protocol was approved in December 2024 by the Ethics Committee of the Universidad Católica Sedes Sapientiae (Approval No. CE-1846). Following coordination with the educational institution, written informed consent was obtained from parents or legal guardians, along with voluntary assent from the adolescents prior to instrument administration. The research was conducted in accordance with the ethical principles of the Declaration of Helsinki (World Medical Association, 2025) and the guidelines of the American Psychological Association (APA, 2017), ensuring confidentiality, anonymity of responses, voluntary participation, and the right to withdraw without consequences at all times.

## 3. Results

### 3.1. Global Network Properties

To characterize the overall topology of the estimated network, we first examined global structural indices. The estimated network exhibited a density of 0.10, identifying 12 connections among the nodes, of which 10 were positive and two were negative, suggesting a relatively parsimonious structure. This low density indicates that only a subset of all possible associations was retained, consistent with a sparse and interpretable network model.

The nodes showed a clear tendency towards clustering, reflected in a high clustering coefficient (Cc) = 0.642, which was greater than expected by chance (Cc_random = 0.375). This pattern suggests the presence of locally interconnected groups of nodes rather than a uniformly connected network. In addition, the average path length was low (APL = 1.78), indicating that nodes are connected through short paths. This implies that activation or change in one node could rapidly propagate through the network. Taken together, these indicators are reflected in a small-world index of S = 1.79, suggesting that the network displays properties typical of a small-world system, characterized by high local integration and efficient global information flow.

### 3.2. Descriptive and Local Network Properties

Before interpreting the network structure, we summarized the distribution of each node. At the descriptive level, the highest mean and standard deviation were observed for self-regulation and external resources (M = 21.54; SD = 7.56), whereas the lowest mean value corresponded to anxiety (M = 6.75). The lowest dispersion was observed for self-confidence and self-trust (SD = 2.79). These results indicate greater variability in regulatory resources and comparatively lower variability in self-confidence. Node redundancy analysis did not suggest the removal of any nodes, indicating that all dimensions provided distinct information and were appropriate to retain in the network.

We next examined local network properties to identify the most influential nodes. With regard to expected influence (EI), the nodes with the highest values were self-regulation and external resources (EI = 1.06), personal competence and tenacity (EI = 0.88), stress (EI = 0.87), and anxiety (EI = 0.82), suggesting that these dimensions were the most strongly connected to the rest of the network and potentially the most impactful in the system. In contrast, happiness exhibited low expected influence (EI = 0.04), indicating a more peripheral position within the relational structure.

Predictability (P) was used to assess how well each node could be explained by its direct neighbors. The nodes with the highest proportion of variance explained were self-regulation and external resources (66.7%), stress (63.9%), depression (63.4%), and anxiety (63.2%), indicating that a substantial part of their variability was embedded in the network structure (Table 2). Lower predictability for happiness (23.3%) further supports its peripheral role. In addition, the Pearson correlation matrix showed both positive and negative correlations, with effect sizes ranging from small to large; these can be consulted in the Supplementary Materials.

### 3.3. Association Between Nodes and Relational Structure

**Figure 1:**
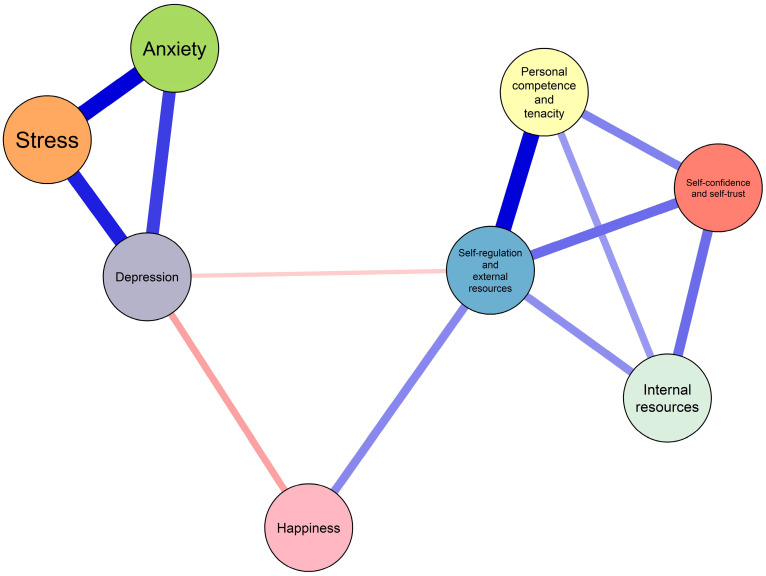
Network structure of anxiety, stress, depression, resilience, and happiness in adolescents estimated using a non-regularized Gaussian Graphical Model (GGM). Blue edges indicate positive associations, and red edges indicate negative associations; edge thickness represents the magnitude of the association. Node size is not scaled to centrality but is presented for visual clarity only.

Figure 1 displays the estimated network structure. The strongest positive connections were observed between stress and anxiety (r = 0.47), self-regulation and external resources, and personal competence and tenacity (r = 0.46), as well as between depression and stress (r = 0.41) and anxiety and depression (r = 0.35). These associations form a closely interconnected core of internalizing symptoms, suggesting that stress, anxiety, and depression are tightly linked within the adolescent mental health system. In contrast, negative associations were identified between depression and happiness (r = −0.18) and between depression and self-regulation and external resources (r = −0.10). These patterns indicate that higher levels of happiness and regulatory resources are associated with lower depressive symptoms.

### 3.4. Network Accuracy and Stability

**Figure 2:**
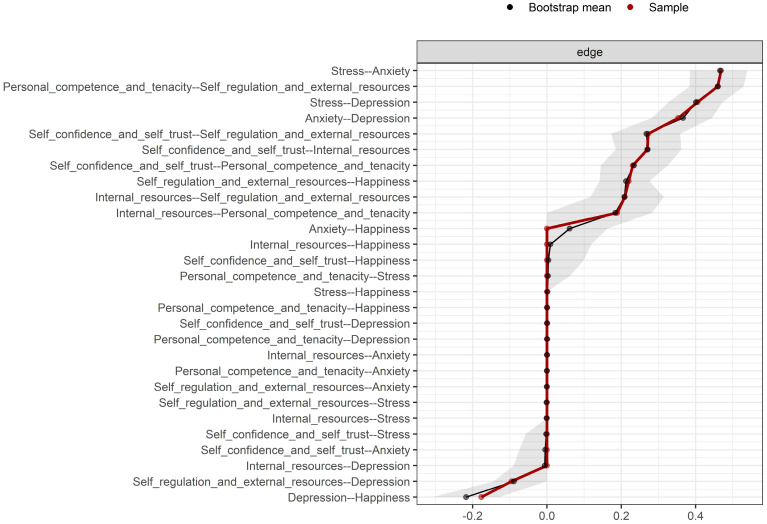
Bootstrapped 95% confidence intervals for all estimated edges in the network. Confidence intervals were obtained through 1000 non-parametric bootstrap resamples. The red dots represent the original sample estimates, whereas the black dots represent the bootstrap means; shaded areas reflect the uncertainty range around each edge.

**Figure 3:**
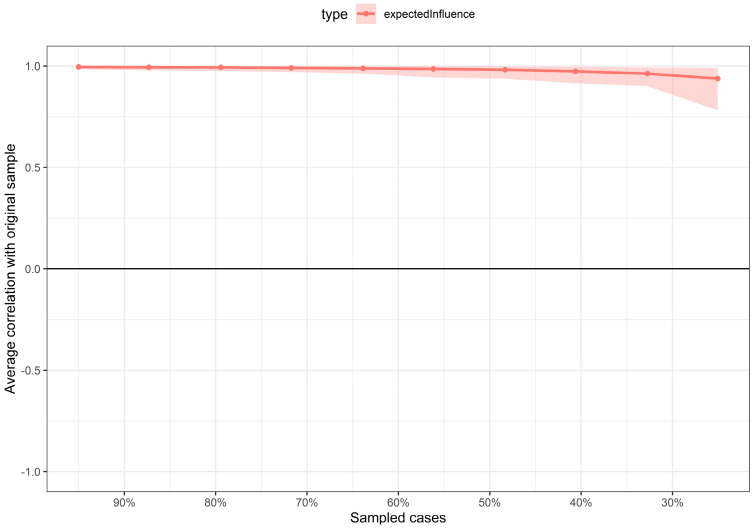
Stability of the expected influence (EI) index assessed using case-dropping subset bootstrapping. The *y*-axis represents the average correlation between the original expected influence values and those obtained after progressively removing increasing proportions of cases. The shaded area represents the variability of the estimate across bootstrap samples.

To evaluate the reliability of the estimated network, we conducted non-parametric bootstrapping. Figure 2 shows the 95% confidence intervals for all edges. Overall, the confidence intervals were narrow and consistent, particularly for stronger connections, supporting the precision of the estimated parameters. In addition, the stability of expected influence was assessed using a case-dropping subset bootstrap. The correlation stability coefficient was high (CS = 0.75; range = 0.673–1.00), exceeding the recommended threshold in the literature, indicating that centrality estimates remained stable even when a substantial proportion of cases was removed (Figure 3).

### 3.5. Network Comparison by Sex

**Figure 4:**
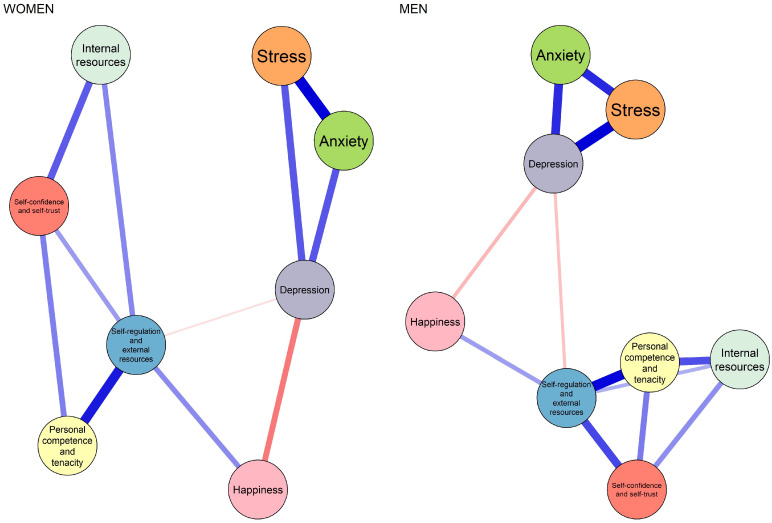
Comparison of network structures by sex (women vs. men). Networks were estimated separately for each group using the same non-regularized GGM procedure. Blue edges indicate positive associations, and red edges indicate negative associations; edge thickness represents the magnitude of the association. The figure illustrates the overall structural similarity between networks prior to formal statistical testing using the Network Comparison Test (NCT).

Figure 4 presents the networks estimated separately for females (n = 280) and males (n = 279). The comparison revealed high structural similarity, with a strong correlation between the weight matrices (r = 0.87). No statistically significant differences were identified in either global network strength (S = 0.003, *p* = 0.985) or overall network structure (M = 0.204, *p* = 0.073). Taken together, these findings indicate that the relational configuration among resilience, happiness, and mental health (anxiety, depression, and stress) is largely comparable across sex. This suggests that, although symptom levels may differ, the underlying pattern of relationships among constructs is structurally similar for males and females. On the other hand, from a frequentist approach, a multivariate analysis of variance (MANOVA) was conducted to examine the effect of sex on self-confidence, internal resources, personal competence and tenacity, self-regulation and external resources, stress, anxiety, depression, and happiness. The results revealed a significant multivariate effect of sex, Pillai’s Trace = 0.095, F (8,550) = 7.20, *p* < 0.001. The Pillai value indicates that approximately 9.5% of the multivariate variance in the set of dependent variables is attributable to sex, suggesting a small to moderate effect size.

## 4. Discussion

The present study reveals a distinctive network topology among adolescents from vulnerable contexts, characterized by a clear functional dichotomy. On the one hand, a dense and self-sustaining pathological core was identified, driven by the feedback loop between stress and anxiety. On the other hand, the protective architecture is not uniform: the self-regulation and external resources dimension emerged as the backbone of the system (highest expected influence and predictability), acting as the primary buffering mechanism against distress. A critical finding is the peripheral structural position of happiness, which—contrary to traditional well-being approaches—appears less integrated into the central symptom machinery; however, this structural peripherality should not be interpreted as evidence of functional or longitudinal irrelevance. Structural invariance by sex suggests that this configuration, in which self-regulation is central and happiness is distal, represents a robust transdiagnostic pattern in this population, independent of sex.

Our findings confirm the existence of a general distress syndrome in adolescents, whereby anxiety, depression, and stress are not isolated entities but components of a dynamically self-reinforcing system (Iannattone et al., 2024; Zhang et al., 2024). However, our network analysis provides a level of structural precision that traditional correlational studies (Moreta-Herrera et al., 2023) often overlook: by isolating unique associations between variables, we reveal the hegemonic role of the direct stress–anxiety edge. This aligns with recent network-based models of psychopathology (He et al., 2023; Wang et al., 2024; Yang et al., 2024), which posit that multidimensional academic and familial stressors function as “bridge” nodes activating anxious symptomatology. In highly vulnerable urban contexts such as North Lima, Peru (Zumba-Tello & Moreta-Herrera, 2022), we suggest that this connection is amplified by developmental sensitivity to environmental threat (Sisk & Gee, 2022), facilitating a rapid loop whereby perceived stress translates directly into anxious activation.

Whereas traditional literature often treats resilience as a monolithic construct (Masten et al., 2021; Mesman et al., 2021), our findings support a more granular and functional perspective. The fact that self-regulation and external resources overwhelmingly surpass personal competence and tenacity in centrality resonates with emerging evidence from affective neuroscience (Nakhostin-Khayyat et al., 2024), which identifies cognitive flexibility and self-regulation as core mechanisms of adaptation, rather than static personality traits. This observation corroborates recent meta-analyses (Pinto et al., 2021; Wen et al., 2023) indicating that resilience is not merely “inner strength” but the capacity to mobilize external resources and regulate emotions in real time. By differentiating these components, our study clarifies that protection against depression is less dependent on dispositional tenacity and more on regulatory capacities that modulate stress reactivity.

The peripheral position of happiness in our network contributes to an ongoing debate on the structure of well-being (Galderisi, 2024; Burns et al., 2022). Although previous studies suggest that subjective happiness is inversely correlated with depression (Extremera & Fernández-Berrocal, 2014; Sotiropoulou et al., 2023), our model indicates that this relationship is distal rather than central. Importantly, given the cross-sectional design, network centrality should not be equated with causal or temporal priority. Our data support the theoretical distinction between hedonic well-being (feeling favorable) and psychological functioning (Blasco-Belled, 2022; Carreno et al., 2021). Moreover, the Subjective Happiness Scale primarily captures a hedonic, evaluative dimension of well-being, which may have limited conceptual overlap with stress-based symptom networks; this measurement focus may partly explain its peripheral structural position. Within the network, happiness appears primarily as an outcome of mental health rather than a bridge capable of disrupting pathological processes. Thus, our findings should be interpreted as reflecting structural positioning within a symptom–resource network, rather than definitive statements about the longitudinal or functional importance of happiness.

Although we critically discuss limitations of traditional latent variable models, the present study operates at the level of theoretically and psychometrically validated subscales rather than individual items. This choice reflects a deliberate trade-off between conceptual granularity and statistical stability. Item-level networks require substantially larger samples to ensure reliable edge estimation and often yield highly complex structures that are difficult to interpret and translate into practice. In contrast, modelling well-established dimensions of resilience, happiness, and psychological distress allows for a more stable and interpretable representation of the mental health system while still capturing direct associations among key psychological domains. Thus, our critique of latent models pertains to their causal assumptions and global-score interpretation, not to the use of validated scales per se.

Furthermore, while network analysis offers important advantages over traditional latent variable models, it is not without methodological controversies. Scholars have raised concerns regarding causal inference in cross-sectional networks, the potential instability of edges under different estimation methods, and the sensitivity of centrality metrics to sampling variability and model specification (Borsboom et al., 2021; Isvoranu & Epskamp, 2023). Although we addressed stability through extensive bootstrapping procedures and employed a non-regularized GGM to reduce shrinkage bias, we acknowledge that alternative estimation approaches (e.g., regularized EBICglasso) could yield different network configurations (Isvoranu & Epskamp, 2023). This underscores the importance of replication, transparency in analytic choices, and cautious interpretation of network parameters as descriptive rather than causal (Borsboom et al., 2021; Jongerling et al., 2023).

From a theoretical standpoint, the centrality of the stress–anxiety pathway and of self-regulation aligns with established models of affective vulnerability and individual differences in stress sensitivity. In particular, research on affective temperaments suggests that baseline emotional reactivity and regulatory styles shape how individuals respond to environmental stressors, influencing both symptom activation and coping trajectories (Iorio et al., 2024). This perspective complements our network findings by indicating that highly reactive temperamental profiles may predispose adolescents to stronger stress–anxiety coupling, whereas more adaptive regulatory temperaments may facilitate the buffering role of self-regulation and external resources within the network.

In addition to structural comparisons, the multivariate analysis revealed significant mean-level sex differences across the set of psychological variables, with sex accounting for a small-to-moderate proportion of the multivariate variance. Although the effect size was modest, this finding aligns with extensive epidemiological literature indicating higher levels of internalizing symptoms—particularly anxiety and depression—among adolescent females compared to males (Piao et al., 2022; Keyes & Platt, 2023). These differences are commonly interpreted within developmental frameworks emphasizing hormonal changes, heightened interpersonal sensitivity, and greater stress reactivity during female adolescence (Sisk & Gee, 2022).

Importantly, the presence of mean-level differences does not contradict the observed structural invariance of the network. Rather, these findings suggest that males and females may differ in overall symptom severity or activation intensity, while maintaining a comparable relational architecture among distress and protective variables. In other words, sex may influence the level at which nodes are activated, but not necessarily the configuration of connections linking them. This distinction between severity and structure is consistent with contemporary network approaches to psychopathology, which differentiate node activation from network topology.

Finally, a crucial contribution of this study is the confirmation of network invariance across sex. Despite epidemiological evidence consistently reporting higher internalizing rates among females (Piao et al., 2022; Keyes & Platt, 2023), our results demonstrate that the functional architecture of the disorder is structurally similar in males and females. This aligns with recent findings in European samples (Sacco et al., 2024) and suggests that prevalence differences are not attributable to distinct psychopathological mechanisms but rather to differences in node activation intensity or sociocultural exposure (Kanougiya et al., 2024). However, network comparison tests do not capture differences in symptom severity, activation thresholds, or developmental timing; therefore, structural invariance can coexist with meaningful sex differences in prevalence or clinical expression.

This supports, rather than guarantees, the case for universal, rather than sex-specific, network-informed preventive strategies in schools.

From a clinical perspective, our findings highlight the centrality of the stress–anxiety edge. Rather than providing definitive treatment prescriptions, these results generate empirically informed hypotheses for intervention design. Our data suggest that techniques targeting acute stress reactivity (e.g., physiological regulation, cognitive reappraisal, and situational coping) may be promising targets rather than focusing solely on broad symptom clusters. Reducing activation at the stress node could, in principle, limit the propagation of distress toward anxiety and depression, a proposition that requires experimental or longitudinal validation.

In the educational domain, the results suggest a potential avenue for refining well-being curricula. Given the peripheral role of happiness observed in the network, programs centered exclusively on motivational talks or generic positive affect promotions might have a limited impact on the broader symptom structure in vulnerable contexts. We hypothesize that schools could benefit from testing strategies that prioritize structured training in self-regulation skills—such as emotional awareness, impulse control, and the strategic use of external resources—as these nodes appeared more central in our model. While our findings identify these competencies as potentially high-impact targets, their causal efficacy on mental health outcomes remains to be confirmed through future experimental or longitudinal intervention studies.

At a policy level, the structural invariance of the network across sex is consistent with the feasibility of universal school-based interventions in middle-income contexts such as Peru. Males and females share a similar relational architecture of distress, suggesting that large-scale programs may be implementable without mandatory sex-differentiated designs, pending further empirical evaluation.

### Limitations

The interpretation of the present findings should consider several limitations. First, the cross-sectional design precludes causal or temporal inference; although network analysis identifies unique associations among variables, it cannot determine whether stress precedes anxiety or vice versa. Longitudinal designs or experience sampling methods (ESM) are needed to examine temporal dynamics.

Second, the use of self-report measures may introduce common method bias and social desirability effects, potentially inflating associations among variables. Future research should incorporate multi-informant data (e.g., parents or teachers) or objective indicators of stress and emotional regulation.

Third, the convenience sample drawn from public schools in North Lima limits the generalizability of the results. The network structure may differ in rural populations, private schools, or clinical samples; thus, replication in diverse contexts is necessary.

Fourth, the network did not include other potentially relevant variables, such as specific traumatic experiences, perceived social support, sleep problems, or academic stressors, which may play important roles in adolescent mental health networks. Future studies should expand the set of nodes to capture a broader range of risk and protective factors.

Fifth, although we employed a non-regularized GGM with rigorous bootstrapping procedures, different network estimation methods (e.g., regularized EBICglasso) could yield slightly different edge patterns. Future studies should replicate these findings using alternative network estimation techniques to strengthen confidence in the robustness of the results.

## 5. Conclusions

This study offers a systemic perspective on adolescent mental health in an urban, lower–middle-income context, showing that psychopathology and psychological resources operate as interdependent components of a single relational system rather than as isolated domains. Grounded directly in our network indicators (edge weights, expected influence, predictability, and stability), our findings yield three empirically supported conclusions.

First, the centrality and strength of the stress–anxiety edge identify stress as a key organizing node of psychological distress in this sample. The strongest connection in the network linked Stress and Anxiety (r = 0.47), and both nodes showed high expected influence (EI = 0.87 and 0.82, respectively) and high predictability (63.9% and 63.2%). These converging indicators suggest that stress is not merely associated with anxiety but occupies a structurally pivotal position within the symptom network, making it a strategic target for prevention and early intervention. Rather than focusing solely on broad diagnostic categories, interventions that reduce acute stress reactivity are likely to disrupt the most influential pathway through which distress propagates.

Second, the pattern of centrality and predictability underscores the primacy of self-regulation and external resources as the main protective component in the system. This dimension exhibited the highest expected influence (EI = 1.06) and the greatest predictability (66.7%), indicating that it was both the most connected node and the one most embedded in the network structure. By contrast, personal competence and tenacity showed lower centrality, and happiness displayed minimal expected influence (EI = 0.04) and low predictability (23.3%), placing it in a peripheral position. These results support a differentiated view of resilience: protection against distress appears to depend more on regulatory capacities and access to external resources than on dispositional traits or on the promotion of positive affect alone. Consequently, prevention programs may be more effective if they prioritize skills for emotional regulation and the strategic use of social and contextual resources.

Third, network stability and structural invariance by sex strengthen the generalizability of these conclusions within this population. The high correlation stability coefficient (CS = 0.75) indicates that centrality estimates were robust to case removal, and bootstrapped confidence intervals were narrow, supporting the reliability of the identified edges. Moreover, network comparison tests revealed no significant differences in global strength (S = 0.003, *p* = 0.985) or overall structure (M = 0.204, *p* = 0.073) between females and males. These findings imply that the relational architecture linking stress, anxiety, depression, resilience, and happiness is structurally similar across sex, even if symptom levels may differ. Therefore, universal, rather than sex-specific, school-based prevention strategies grounded in self-regulation training are empirically justified.

Overall, the present results suggest that adolescent mental health in this context is best understood as a dynamic network in which stress and self-regulation play central roles, whereas happiness functions primarily as an outcome rather than a driver of change. This network-informed perspective provides a more precise map of potential intervention targets than traditional global-score approaches, with direct implications for clinical practice, educational programming, and public mental health policy in resource-constrained settings.

## Figures and Tables

**Table 1:** Sociodemographic characteristics of the sample.

Variable	Categories	*n*	%
Sex	Male	279	49.91
	Female	280	50.09
Age (years)	Mean ± SD	14.72 ± 1.43	
Peer relationship	Poor	20	3.58
	Fair	269	48.12
	Good	270	48.3
Family relationship	Poor	12	2.15
	Fair	211	37.75
	Good	336	60.11
Study resources	No	53	9.48
	Yes	506	90.52
Recreational activities	No	101	18.07
	Yes	458	81.93
Psychological treatment	No	285	50.98
	Yes	274	49.02

Note. *n* = frequency; % = percentage; SD = standard deviation. Availability of study resources was assessed using a dichotomous self-report item.

**Table 2:** Descriptive measures and local network properties.

Nodes	Descriptive				Local Properties	
	M	SD	g_1_	g_2_	EI	P
Self-confidence and self-trust	7.96	2.79	−0.54	−0.23	0.77	54.10%
Internal resources	11.64	4.12	−0.18	−0.27	0.67	49.50%
Personal competence and tenacity	11.33	4.28	−0.09	−0.41	0.88	62.00%
Self-regulation and external resources	21.54	7.56	−0.27	−0.29	1.06	66.70%
Stress	7.46	4.34	0.5	−0.42	0.87	63.90%
Anxiety	6.75	4.66	0.63	−0.34	0.82	63.20%
Depression	7.11	5.13	0.65	−0.34	0.49	63.40%
Happiness	19.08	4.59	−0.26	0.06	0.04	23.30%

Note. M = mean, SD = standard deviation, g_1_ = skewness, g_2_ = kurtosis, EI = expected influence, P = predictability.

## Data Availability

The data presented in this study are available upon request from the corresponding author due to privacy issues. All individuals and groups acknowledged are referred to in a collective and anonymous manner; no personally identifiable information is disclosed, and participation and consent procedures were covered under the general informed consent approved by the institutional ethics committee.

## Supplementary Materials

The following supporting information can be downloaded at: https://www.mdpi.com/article/10.3390/ejihpe16020031/s1, Table S1: Pearson correlation matrix of all study variables.
