# Supplementary material for: Mapping Anxiety, Stress, Depression, Resilience and Happiness in the Adolescent Population: A Network Analysis and Comparison by Sex
Source: Eur J Investig Health Psychol Educ. 2026 Feb 19;16(2):31. doi: 10.3390/ejihpe16020031 (PMC12939747; doi:10.3390/ejihpe16020031)
Supplement: Supplementary file 1 [file ejihpe-16-00031-s001.zip › ejihpe-4115679-supplementary.pdf]

Supplementary material

Mapping anxiety, stress, depression, resilience and happiness in the adolescent population: a network analysis and comparison by sex

Correlation analysis

According to Table 3, the correlations among the psychological variables were both positive and negative, with effect sizes ranging from small to large.

Table S1. Pearson correlation matrix

Variable	Self-confidence and self-trust	Internal resources	Personal competence and tenacity	Self-regulation and external resources	Stress	Anxiety	Depression	Happines
**Self-confidence and self-trust**	-							
**Internal resources**	0.623*	-						
**Personal competence and tenacity**	0.654*	0.623*	-					
**Self-regulation and external resources**	0.673*	0.637*	0.750*	-				
**Stress**	-0.149*	-0.130*	-0.141*	-0.225*	-			
**Anxiety**	-0.141*	-0.099*	-0.138*	-0.196*	0.756*	-		
**Depression**	-0.197*	-0.198*	-0.220*	-0.319*	0.721*	0.708*	-	
**Happiness**	0.283*	0.274*	0.296*	0.385*	-0.219*	-0.162*	-0.367*	-

Note. p < 0.001*
